# Developmental loss of neurofibromin across distributed neuronal circuits drives excessive grooming in *Drosophila*

**DOI:** 10.1371/journal.pgen.1008920

**Published:** 2020-07-22

**Authors:** Lanikea B. King, Tamara Boto, Valentina Botero, Ari M. Aviles, Breanna M. Jomsky, Chevara Joseph, James A. Walker, Seth M. Tomchik

**Affiliations:** 1 Department of Neuroscience, The Scripps Research Institute, Jupiter, Florida, United States of America; 2 Honors College, Florida Atlantic University, Jupiter, Florida, United States of America; 3 Center for Genomic Medicine, Massachusetts General Hospital, Harvard Medical School, Cambridge, Massachusetts, United States of America; UCSB, UNITED STATES

## Abstract

Neurofibromatosis type 1 is a monogenetic disorder that predisposes individuals to tumor formation and cognitive and behavioral symptoms. The neuronal circuitry and developmental events underlying these neurological symptoms are unknown. To better understand how mutations of the underlying gene (*NF1*) drive behavioral alterations, we have examined grooming in the *Drosophila* neurofibromatosis 1 model. Mutations of the fly *NF1* ortholog drive excessive grooming, and increased grooming was observed in adults when Nf1 was knocked down during development. Furthermore, intact Nf1 Ras GAP-related domain signaling was required to maintain normal grooming. The requirement for Nf1 was distributed across neuronal circuits, which were additive when targeted in parallel, rather than mapping to discrete microcircuits. Overall, these data suggest that broadly-distributed alterations in neuronal function during development, requiring intact Ras signaling, drive key Nf1-mediated behavioral alterations. Thus, global developmental alterations in brain circuits/systems function may contribute to behavioral phenotypes in neurofibromatosis type 1.

## Author summary

Neurofibromatosis type 1 results in increased susceptibility to cognitive and behavioral symptoms, such as attention-deficit/hyperactivity disorder. This suggests that the underlying genetic mutations affect neuronal function. Where and when these effects occur in the brain is not understood. We approached these questions in the genetically-powerful fly model, examining how loss of the *NF1* gene affects a complex motor behavior, grooming. Loss of *NF1* during brain development caused excessive grooming in adult animals. This suggests that the gene plays a key role in brain development, which may be important for treatment of the disorder. Further, we found that a specific signaling pathway, Ras, is involved in regulating this grooming behavior. Finally, we mapped where in the brain the neurofibromin (Nf1) protein is required to maintain normal levels of grooming. The data suggested that it is required across many neurons, rather than in small subsets. When Nf1 was knocked down in smaller subsets, there was no effect, but if two subsets were added together, an effect was produced. This means that Nf1 regulates the function of many neurons and brain regions to influence complex behaviors.

## Introduction

Many genetic disorders affect cognitive and behavioral function in humans. Among these, neurofibromatosis type 1 (NF1) affects approximately 1 in 3000 people, making it among the most common monogenetic disorders associated with cognitive dysfunction [1,2]. While its core symptoms include neurofibromas, optic gliomas, and café au lait spots, the disorder also predisposes individuals to cognitive or behavioral symptoms such as hyperactivity, problems with attention, disrupted sleep, repetitive behaviors, and difficulty with social encounters [1–9]. Along these lines, NF1 increases risk for attention-deficit/hyperactivity disorder [1,2,6] and autism spectrum disorder [4,5,7,9,10]. These cognitive and behavioral symptoms affect quality of life and are considered major contributors to lifetime morbidity in NF1 patients [1,10,11]. NF1 is caused by a mutation in the *NF1* gene encoding the neurofibromin (Nf1) protein. Neurofibromin acts as a GTPase-activating protein (GAP) for Ras, thus reducing active Ras signaling [12–14]. In addition to this major role in Ras signaling, neurofibromin has also been implicated in G protein-coupled receptor signal transduction, modulation of cAMP levels, and dopaminergic circuit function [15–21]. These pathways modulate both developmental processes, such as cell proliferation, migration, fate specification, apoptosis, and morphology, as well as cellular physiology and plasticity in adults [22,23].

How loss of neurofibromin drives behavioral alterations, via effects on neuronal circuits, is a major question. NF1 phenotypes have been associated with a variety of cell types. For example, tumors in NF1 arise from multiple cell types: optic gliomas originate from astroglia [24], while neurofibromas originate from Schwann cells [25]. In some cases, behavioral phenotypes have been mapped to discrete neuronal populations in the brain. Learning deficits in *Nf1* mutant mice stem from the loss of the gene in GABAergic inhibitory interneurons but not excitatory neurons or glia [26]. Motor and depression-like phenotypes in a mouse model map to dopamine receptor D1 positive neurons in the striatum [27]. In *Drosophila*, *Nf1* mutants exhibit olfactory learning deficits that map to a subset of neurons in the mushroom bodies, important structures for learning and memory [28]. However, in other cases, *Nf1* mutant phenotypes map only to broader sets of cells. For instance, in flies, a *Nf1*-dependent body size phenotype maps only broadly to neurons [29]. Thus, localization, scope, and neurochemical identity of the neuronal circuit underlying cognitive and behavioral phenotypes in NF1 are variable and unclear. To understand how the loss of Nf1 affects neuronal circuits underlying motor behaviors, through development and into adulthood, the present study examines the biological underpinnings of repetitive aberrant grooming in the *Drosophila* NF1 model.

## Results

### The ventral nervous system is a major locus of Nf1-sensitive grooming circuits

**Fig 1:**
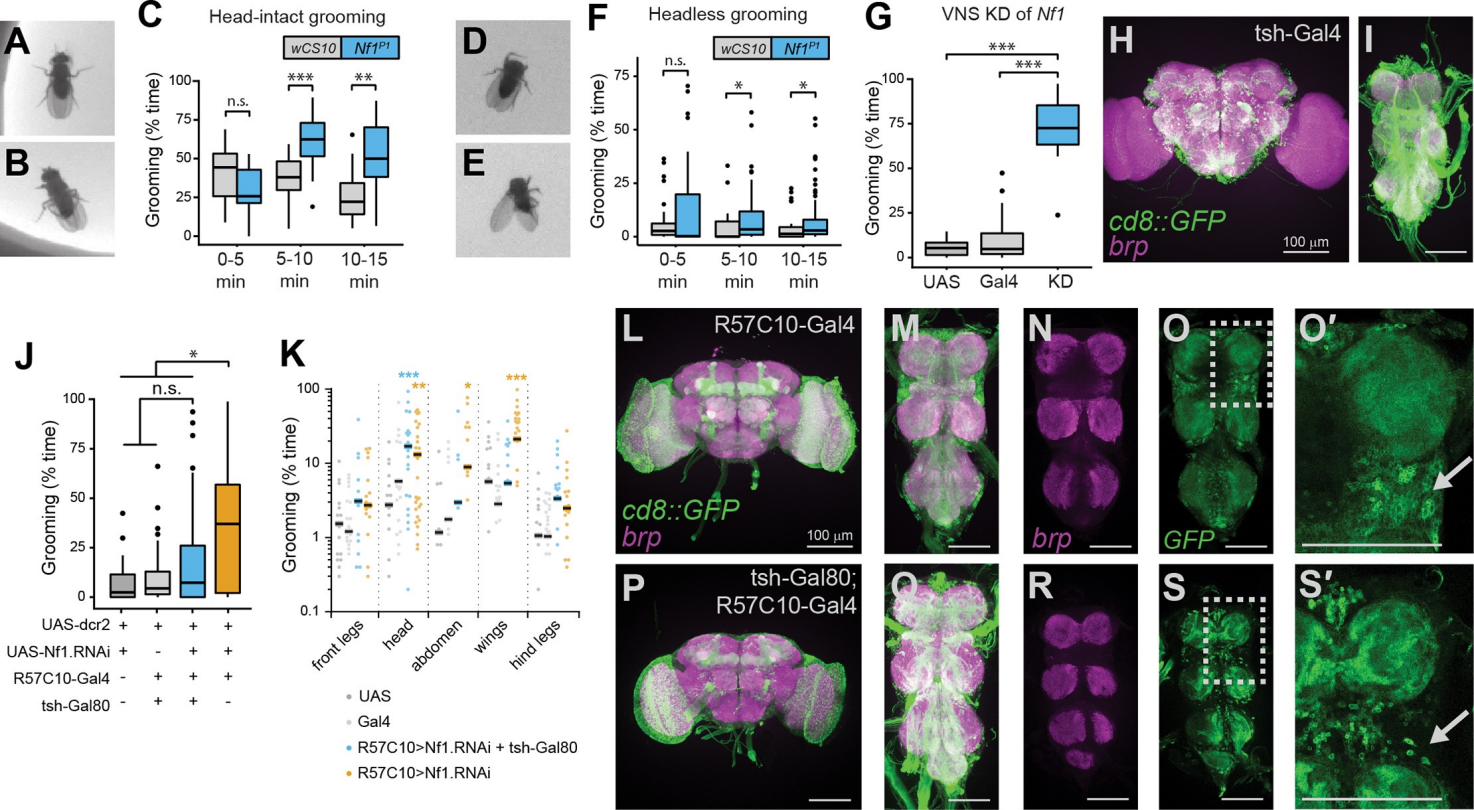
Neurons in the brain and ventral nervous system that modulate grooming are sensitive to loss of Nf1. (A) An intact fly grooming its anterior (prothoracic) legs. (B) An intact fly grooming its posterior (metathoracic) legs. (C) Grooming duration in intact *Nf1*^P1^ vs. *wCS10* control flies at 0, 5, and 10 min after introduction to the chamber. n = 18; **p < 0.01, ***p < 0.001 (Wilcoxon rank-sum test). (D) Grooming movement directed toward the anterior body (missing head) in a decapitated fly. (E) A headless fly grooming its hind (metathoracic) legs. (F) Grooming duration in decapitated flies. n = 25–26; *p < 0.05 (Wilcoxon rank-sum test). (G) Grooming duration in flies with Nf1 knockdown in tsh-Gal4+ neurons. n = 16; p < 0.001 (Kruskal-Wallis); ***p < 0.001 (Dunn/Sidak). (H) Maximum-intensity projection showing tsh>UAS-mCD8::GFP (green) and bruchpilot (brp; magenta) in the central brain. Scale bar = 100 μm. (I) VNS expression of tsh and brp, as in panel H. (J) Grooming duration with pan-neuronal knockdown (KD) of Nf1 via the R57C10-Gal4, with or without the tsh-Gal80 repressor. n = 40–42; p < 0.001 (Kruskal-Wallis); *p < 0.05 (Dunn/Sidak). (K) Grooming duration from the same data set in panel J, graphed by body part groomed. Individual data points are graphed (zero values are not plotted), with the mean shown as a line. Stars represent significance re: UAS control. (L) Maximum-intensity projection of anti-GFP (green) and anti-brp (magenta) immunostaining in the central brain of an R57C10-Gal4 > UAS-mCD8::GFP fly. Scale bar = 100 μm. (M) R57C10-Gal4 expression pattern in the VNS, imaged as in panel K. (N) Single z-plane showing anti-brp neuropil staining from the VNS in panel L. (O) GFP staining from the VNS plane in panel L. (O’) Expanded detail from the dashed box in panel N. Arrow points to area of dense somata. (P) Maximum-intensity projection of the brain of an R57C10-Gal4 > UAS-mCD8::GFP, UAS-tsh-Gal80 fly. (Q) Expression pattern in the VNS of the same fly shown panel K. (R) Single z-plane showing anti-brp neuropil staining from the VNS in panel P. (S) GFP staining from the VNS plane in panel Q. (S’) Expanded detail from the dashed box in panel N. Arrow points to area of reduced somata density by tsh-Gal80 repression.

Adult flies bearing *Nf1* loss of function mutations and RNAi knockdown exhibit excessive spontaneous grooming, reflecting an increase in grooming frequency and duration, due to loss of Nf1 in neurons [30]. The fly central nervous system (CNS) consists of a central brain and a ventral nervous system (VNS) that, like the mammalian spinal cord, is necessary for nearly all motor output. To gain insight into the neuronal circuits mediating this increase in grooming, we first parsed the roles of neurons in the brain from those in the VNS. Descending neurons send projections from the brain to the VNS and initiate a range of behaviors including grooming [31–33]. To test whether loss of Nf1 increased grooming via neurons originating in the brain, we decapitated *Nf1*^P1^ mutants and observed grooming in an open field. *Nf1*^P1^ mutants harbor a large deletion in the *NF1* locus and express no functional protein [16]. Because decapitation requires anesthesia, we first tested whether intact flies groom normally after anesthesia. Normal grooming behaviors were observed after anesthesia, including grooming of the head (Fig 1A) and abdomen (Fig 1B). As previously reported [34,35], decapitated flies survive until desiccated and typically stand still or groom. During the first five minutes after transfer to an open field chamber, *Nf1*^*P1*^ mutants and genetic controls groomed at indistinguishable durations (Fig 1C). However, after 5 min of acclimation to the open field, *Nf1*^*P1*^ mutants groomed significantly longer than controls (Fig 1C). This is consistent with previous results in which *Nf1* mutants groomed more than control flies after an acclimation period [30]. In addition to grooming intact posterior body parts (Fig 1E), decapitated flies made grooming-like front leg sweeps toward the location of the missing head, suggesting that the head grooming motor pattern can be activated in the absence of the actual head and brain (Fig 1D). Similar to intact animals, decapitated *Nf1* mutants and genetic controls groomed for equivalent durations during the first 5 minutes in the open field (Fig 1F). However, after acclimation, decapitated *Nf1* mutants exhibited a significant increase in grooming relative to genetic controls (Fig 1F). Thus, overall, *Nf1* mutants exhibited elevated levels of grooming in the absence of a central brain, preliminarily pointing toward VNS circuits as a major locus of Nf1 sensitivity.

To further test the role of Nf1 in VNS circuits with a genetic approach, we turned to the Gal4/UAS system and RNAi-mediated knockdown of Nf1 [36]. Using the tsh-Gal4 driver [37] to knock down Nf1 preferentially in the VNS, we observed significantly elevated grooming compared to genetic controls (Fig 1G). To validate the VNS specificity of tsh-GAL4, we used the driver to express UAS-mCD8::GFP and found relatively sparse expression of GFP in the brain (Fig 1H) and widespread labeling of neuropil and cell bodies in the VNS (Fig 1I). We next used the converse approach to determine whether VNS knockdown of Nf1 is necessary for excessive grooming, using a tsh-GAL80 transgene to subtract a subset of VNS neurons that would otherwise be targeted by a pan-neuronal driver. Flies that were spared Nf1 knockdown in the VNS groomed significantly less than pan-neuronal Nf1 knockdown flies–their total grooming did not significantly differ from genetic controls lacking the driver or effectors (Fig 1J). However, analysis of the grooming of individual body parts revealed a more nuanced picture. Pan-neuronal knockdown significantly increased grooming of the head, abdomen, and wings (Fig 1K). Addition of the tsh-Gal80 transgene eliminated this excess grooming in the wings and abdomen, suggesting that the localization of Nf1-sensitive neurons driving wing and abdomen grooming is biased toward the VNS. However, head grooming remained elevated (Fig 1K), suggesting that some of the Nf1-sensitive neurons driving head grooming localize to the brain. Using immunohistochemistry, we compared brain and VNS labeling of R57C10-Gal4 (Fig 1L and 1M) and tsh-GAL80;R57C10-Gal4 (Fig 1P and 1Q). We observed a loss of VNS cell body staining in flies carrying tsh-GAL80 (Fig 1S) compared to pan-neuronal R57C10-Gal4 labeling (Fig 1O), consistent with previous observations [38]. Together, these results suggest that Nf1-sensitive grooming circuits are distributed across both the brain and VNS, with the VNS being a key site for Nf1-sensitive grooming neurons.

### Neurochemical identity of Nf1-sensitive grooming neurons

**Fig 2:**
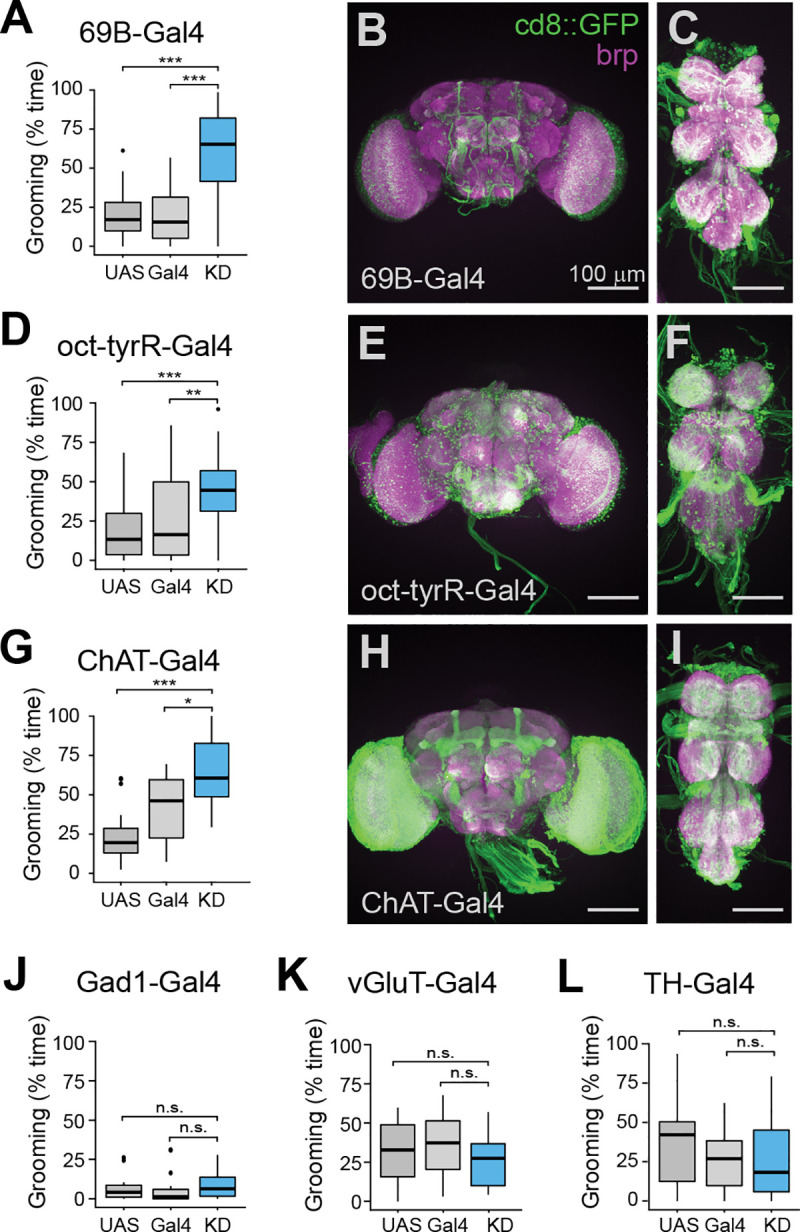
Loss of Nf1 in broad sets of excitatory neurons increases grooming. (A) Grooming duration with Nf1 knockdown (KD) in 69B-Gal4+ neurons. n = 20; p < 0.001 (Kruskal-Wallis). (B) Maximum-intensity projection of 69B-Gal4 expression (green) and anti-brp immunostaining (magenta) in the brain. Scale bar = 100 μm. (C) VNS expression of 69B as in panel B. (D) Knockdown of Nf1 in oct-tyrR-Gal4+ neurons. n = 20; p < 0.001 (Kruskal-Wallis). (E) Brain expression of the oct-tyrR-Gal4 driver. (F) VNS expression of the oct-tyrR-Gal4 driver. (G) Knockdown of Nf1 using the ChAT-T2A-Gal4 driver. n = 20; p < 0.001 (Kruskal-Wallis). (H) Brain expression of the ChAT-T2A-Gal4 driver. (I) VNS expression of the ChAT-T2A-Gal4 driver. (J) Knockdown of Nf1 in Gad1-Gal4+ neurons. n = 18 each; p = 0.23 (Kruskal-Wallis). (K) Knockdown of Nf1 in vGluT-Gal4+ neurons. n = 20; p = 0.45 (Kruskal-Wallis). (L) Knockdown of Nf1 in TH-Gal4+ neurons. n = 20; p = 0.6 (Kruskal-Wallis). *p < 0.05, **p < 0.01, ***p < 0.001 (Dunn/Sidak).

To determine whether any major cell types are differentially associated with the excessive grooming phenotype, we knocked down Nf1 in defined neuronal subsets. A driver previously associated with a *Nf1* body size phenotype, 69B-Gal4 [29], increased grooming when used to knock down *Nf1* (Fig 2A). This driver labels the brain sparsely (Fig 2B) and the VNS more strongly (Fig 2C). Knocking down Nf1 in cholinergic neurons, the primary excitatory neuronal population in the fly nervous system, with ChAT-Gal4 also resulted in excessive grooming compared to genetic controls (Fig 2G). This driver labels neurons broadly across the brain (Fig 2H) and VNS (Fig 2I). In contrast, knockdown of Nf1 in neurons expressing the other major neurotransmitters GABA (Gad1-Gal4) or glutamate (vGluT-Gal4) did not produce any differences in grooming (Fig 2J and 2K). Likewise, Nf1 knockdown in dopaminergic neurons using TH-Gal4 did not influence grooming behavior (Fig 2L). However, the oct-tyrR-Gal4 [39] driver that labels a subset of neurons that express the oct-tyrR receptor, produced excessive grooming when used to knock down Nf1 (Fig 2D). This driver labels a broadly-distributed but relatively sparse population of neurons in the brain (Fig 2H) and VNS (Fig 2I). The oct-tyrR receptor responds to both octopamine and tyramine [40], suggesting that monoaminergic sensitive oct-tyrR+ neurons may be a Nf1-sensitive population that regulates grooming levels. Collectively, these data suggest that the population of neurons associated with elevated grooming is broadly distributed, biased toward the VNS, excitatory, cholinergic, and oct-tyrR+.

### Excessive grooming involves the Nf1 GAP-related domain

**Fig 3:**
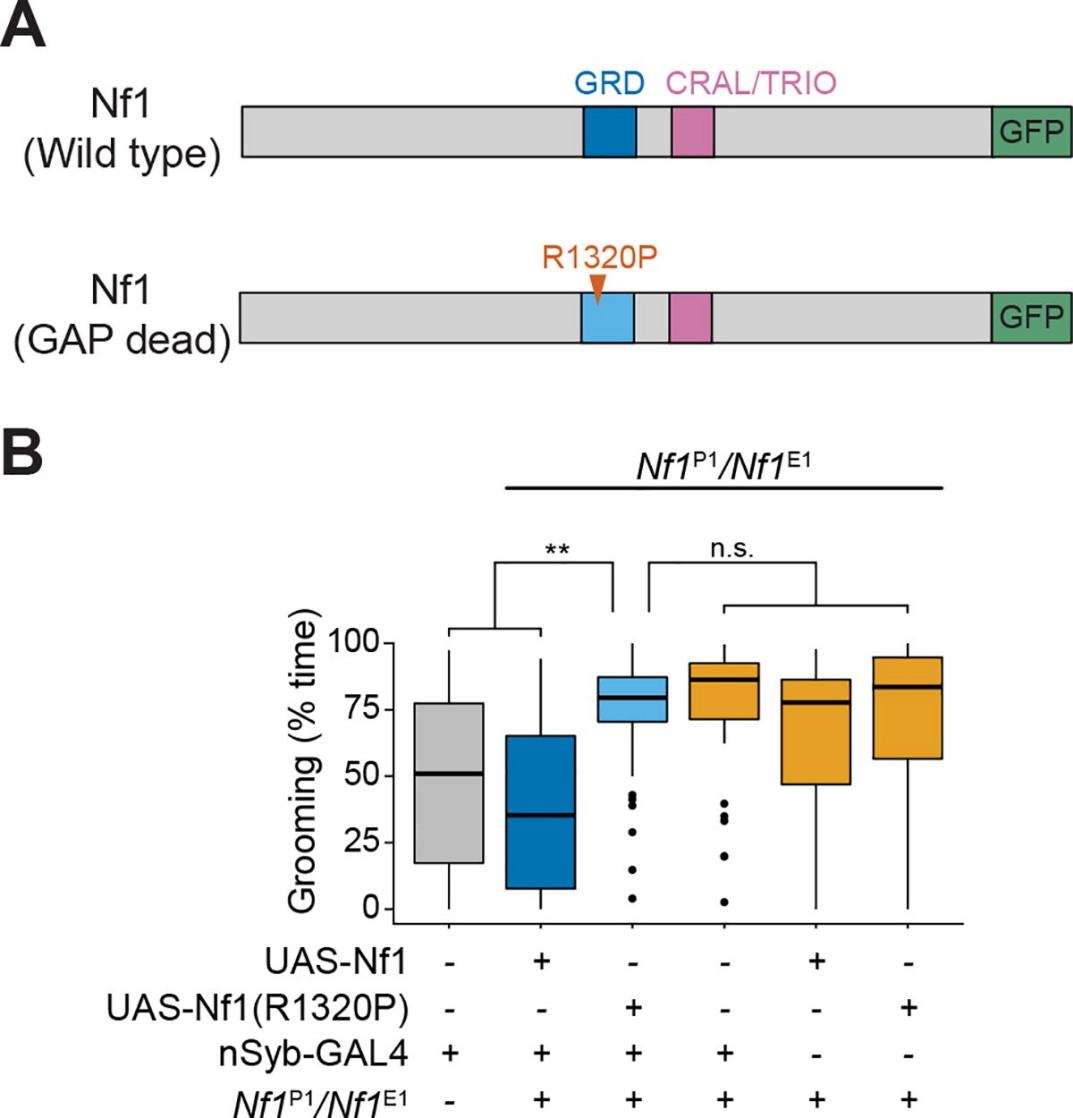
Functional Nf1 GAP-related domain (GRD) is required to maintain normal grooming frequency. (A) Diagram of the wild type (top) and mutated (bottom) Nf1 rescue constructs. Major protein domains, the R1320P mutation, and GFP C-terminal fusions are depicted. (B) Grooming durations for (left to right): (1) nSyb-Gal4 genetic control, and *Nf1*^P1^/*Nf1*^E1^ mutants expressing (2) wild-type UAS-Nf1 rescue, (3) UAS-Nf1^R1320P^ rescue, (4) nSyb-Gal4 alone, (5) UAS-Nf1 alone, and (6) UAS-Nf1^R1320P^ alone. n = 40; p < 0.001, Kruskal-Wallis; **p < 0.01, n.s.: not significant (Dunn/Sidak).

The most well-described biochemical function of Nf1 is its conserved Ras-GAP activity, inhibiting Ras [12,41]. However, it also affects other signaling cascades via direct and putative indirect interactions, such as G protein-coupled receptor signaling and cAMP/PKA regulation [16,17,19,27,42–46]. To begin probing the critical downstream effectors regulating the grooming phenotype, we used the Gal4-UAS system to express wild-type and GAP-related domain (GRD) mutant Nf1 transgenes in an *Nf1* mutant background. For this experiment, we used a heteroallelic *Nf1*^P1^/*Nf1*^E1^ mutant. The *Nf1*^E1^ allele is a nonsense mutation that truncates the protein upstream of the GRD [29]. This heteroallelic combination, chosen to circumvent lethality of the E1 mutation and facilitate introduction of transgenic rescue constructs, exhibited excessive grooming, similar to the homozygous *Nf1*^P1^ mutants and pan-neuronal Nf1 RNAi (Fig 3B). In this background, we pan-neuronally expressed either a wild-type Nf1 transgene (Fig 3A) or one containing a point mutation (R1320P) in the Ras GAP-related domain (GRD), which impairs its catalytic activity (Fig 3A). The R1320P mutation is modeled on the orthologous human neurofibromatosis 1 patient-derived R1276P mutation. This mutation in the “arginine finger” of the GRD prevents Nf1 from binding Ras and reduces GAP activity >8000 fold [29,47]. Expressing the wild-type Nf1 transgene reduced grooming to control levels (Fig 3B). However, the Nf1^R1320P^ transgene failed to rescue, as flies expressing it groomed significantly more than both wild-type controls and flies expressing the wild-type rescue construct (Fig 3B). Thus, the ability of Nf1 to regulate Ras is crucial to maintaining normal grooming activity.

### Nf1 regulates adult grooming via a developmental critical window

**Fig 4:**
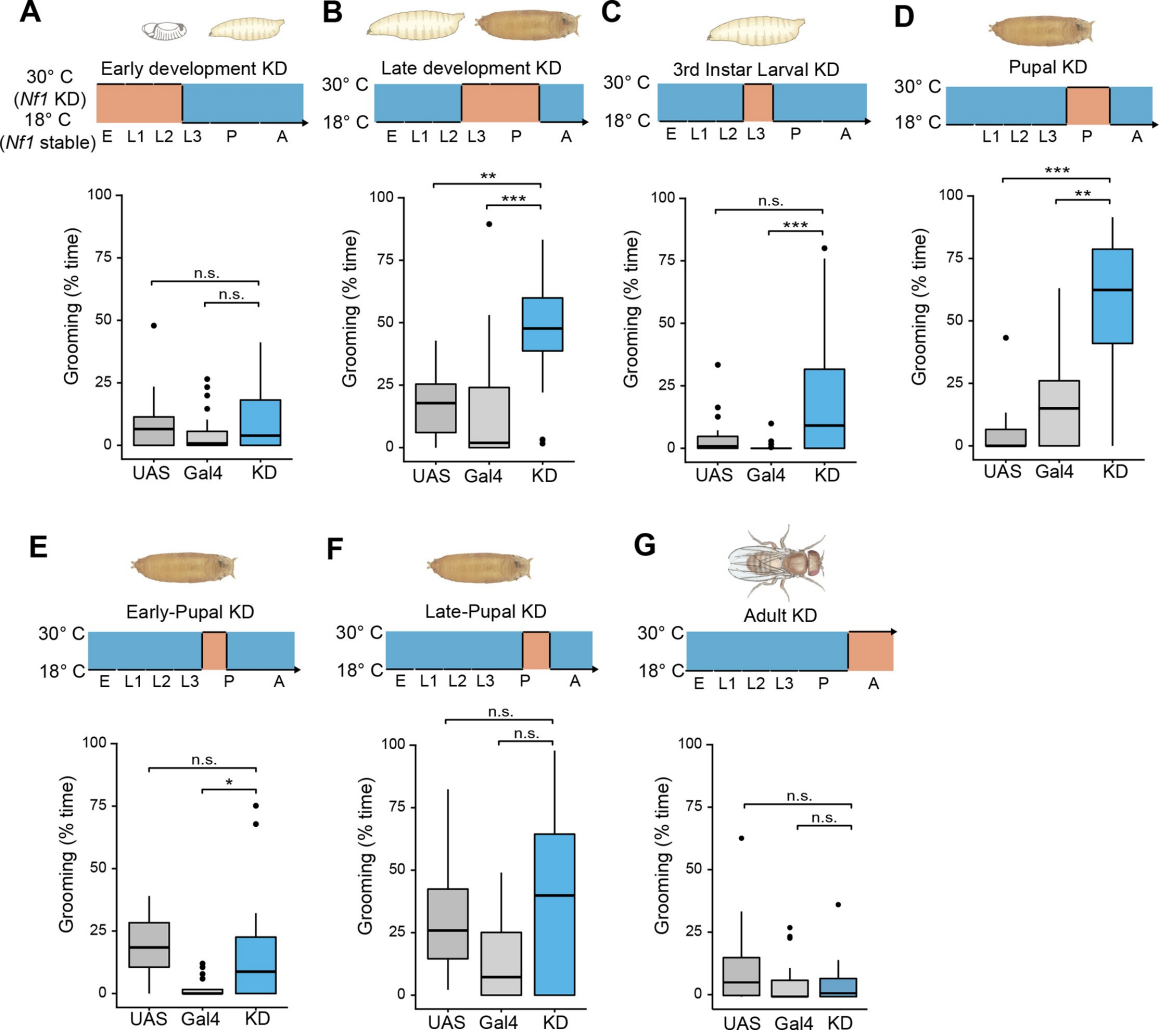
Excessive grooming results from the loss of Nf1 during a critical developmental window. Gal80^ts^ was used to restrict knockdown (KD) of Nf1 with RNAi to different time windows during development and adulthood. (A-G) In each panel, elav-Gal4 > UAS-Nf1 RNAi, tub-Gal80^ts^ (knockdown [KD]) is compared to UAS-Nf1 RNAi (UAS) and elav-Gal4 and tub-Gal80^ts^ (Gal4) controls. n = 20. *p < 0.05, **p < 0.01, ***p < 0.001 (Dunn-Sidak). (A) Knockdown during embryo through larval L2 stage. p = 0.37 (Kruskal-Wallis). (B) Larval L3 and pupal stages. p < 0.001 (Kruskal-Wallis). (C) Larval L3 stage. p < 0.001 (Kruskal-Wallis). (D) Pupal stage. p < 0.001 (Kruskal-Wallis). (E) Early pupal stage. p < 0.001 (Kruskal-Wallis). (F) Late pupal stage. p = 0.05 (Kruskal-Wallis). (G) Knockdown in adulthood, following eclosion. p = 0.1 (Kruskal-Wallis).

To determine whether excessive grooming reflects developmental effects or an ongoing requirement for Nf1 in adult neurons, we used the Gal80^ts^ conditional expression system [48]. A ubiquitously expressed temperature-sensitive GAL80 (tub-GAL80^ts^) was used to control Gal4-UAS activity driving Nf1 RNAi. We first raised embryos or larvae at the permissive temperature (18°C) to prevent GAL4/UAS-mediated Nf1 knockdown during development, and subsequently placed the eclosed adults at the restrictive temperature (30°C), knocking down Nf1 only during adulthood. Adult-specific Nf1 knockdown did not produce a significant effect on grooming (Fig 4G). In contrast, conditional knockdown of Nf1 during the late larval (3^rd^ instar) and pupal stages (Fig 4B), but not early development (through 2^nd^ instar) (Fig 4A), produced excessive grooming in adulthood. Reducing the duration of Nf1 knockdown, we found that knockdown during the pupal stage caused significantly higher levels of grooming over genetic controls (Fig 4D), whereas knockdown during only the 3^rd^ instar larval stage did not significantly change grooming from both genetic controls (Fig 4C). Subdividing Nf1 knockdown within the pupal stage into halves produced no significant effect, relative to both controls, in either half (Fig 4E and 4F). Therefore, we conclude that the pupal stage is a critical developmental window when Nf1 is required for the development of circuits that maintain appropriate levels of grooming activity in adults.

### Developmental pattern of Nf1-sensitive neurons during the pupal stage

**Fig 5:**
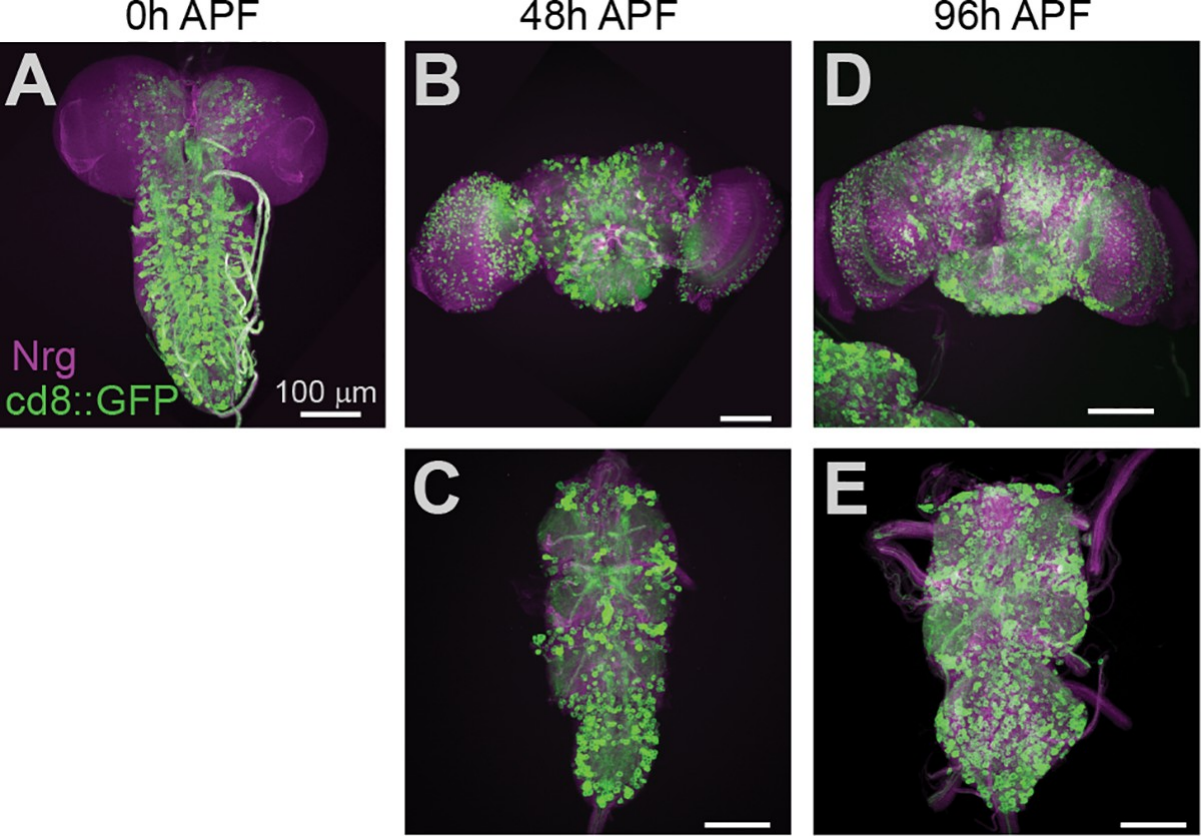
Octopamine-tyramine receptor+ cells are abundant throughout development. (A) Maximum-intensity projection of oct-tyrR+ neurons (green) and anti-neuroglian (Nrg) (magenta) immunostaining in the 0-hr pupal nervous system. (B) 48-hr pupal brain. (C) 48-hr pupal VNS. (D) 96-hr pupal brain. (E) 96-hr pupal VNS. APF: after puparium formation.

Among the Gal4 lines that produced an increase in grooming when knocking down Nf1, the oct-tyrR-Gal4 labels a neurochemically discrete population of neurons, which have been associated with locomotor control [49,50]. Gal4 expression patterns can shift during development [51]. As Nf1 is expressed broadly in the nervous system [29], this opens at least two potential mechanisms for the developmental regulation of grooming: the phenotype could be due to dynamic knockdown of Nf1 caused by changes in driver expression over time, or the cellular requirement for Nf1 could change over time despite relatively stable RNAi expression. To discriminate between these possibilities, we examined the expression patterns of the oct-tyrR-Gal4 driver during the pupal stage. Using immunohistochemistry, we visualized oct-tyrR+ neurons at the beginning (Fig 5A), middle (Fig 5B and 5C), and end (Fig 5D and 5E) of the pupal stage. oct-tyrR+ neurons were well represented throughout development and broadly innervate neuropil in the brain and VNS.

### Additive influence of microcircuits is required to express hyperactive grooming

To test smaller subsets of neurons for sensitivity to Nf1 knockdown, we screened a subset of 212 genomic enhancer-driven Gal4 lines that exhibit sparse expression in the VNS [52]. In an initial screen, each Gal4 line was used to knock down Nf1 and grooming was scored (n = 10 each). These lines exhibited a range of grooming frequencies, from nearly zero to over 50% median time spent grooming (S1 Fig). The lines with the highest absolute grooming frequencies (> 40%) were selected for follow-up testing, which involved comparing the knockdown genotype (Gal4>UAS-Nf1) with two genetic controls (Gal4/+ and UAS-Nf1/+). None of the lines selected for follow-up testing exhibited grooming significantly and consistently elevated relative to both controls (S2 Fig). Among lines that did not reach threshold for follow-up were lines that express in sensory neurons across the body and have been previously shown to elevate grooming when stimulated (R52A06-, R30B01-, and R81E10-GAL4) [35]. They did not recapitulate the excessive grooming phenotype when used to knock down Nf1 (S1 Fig). Similarly, several other neuronal subsets that drive grooming when stimulated (R53A06 and R50B07: wing grooming, R23A07: eye/head grooming) [53], did not recapitulate the pan-neuronal Nf1 phenotype (S1 Fig). Thus, elevated grooming was only observed when knocking down Nf1 with drivers that exhibit broad expression patterns: pan-neuronal, 69B, ChAT, and oct-tyrR. We cannot rule out the possibility that our screen missed a particular sparse set of neurons that would have produced a strong phenotype. Nonetheless, we reasoned that Nf1 may be required across distributed neurons for the development of normal grooming patterns, and consequently, knocking Nf1 down in more restricted neuronal subsets was insufficient to recapitulate the phenotype. If this was the case, some of the sparsely-labeled lines from our screen would be expected to function additively, further elevating grooming frequency when combined.

**Fig 6:**
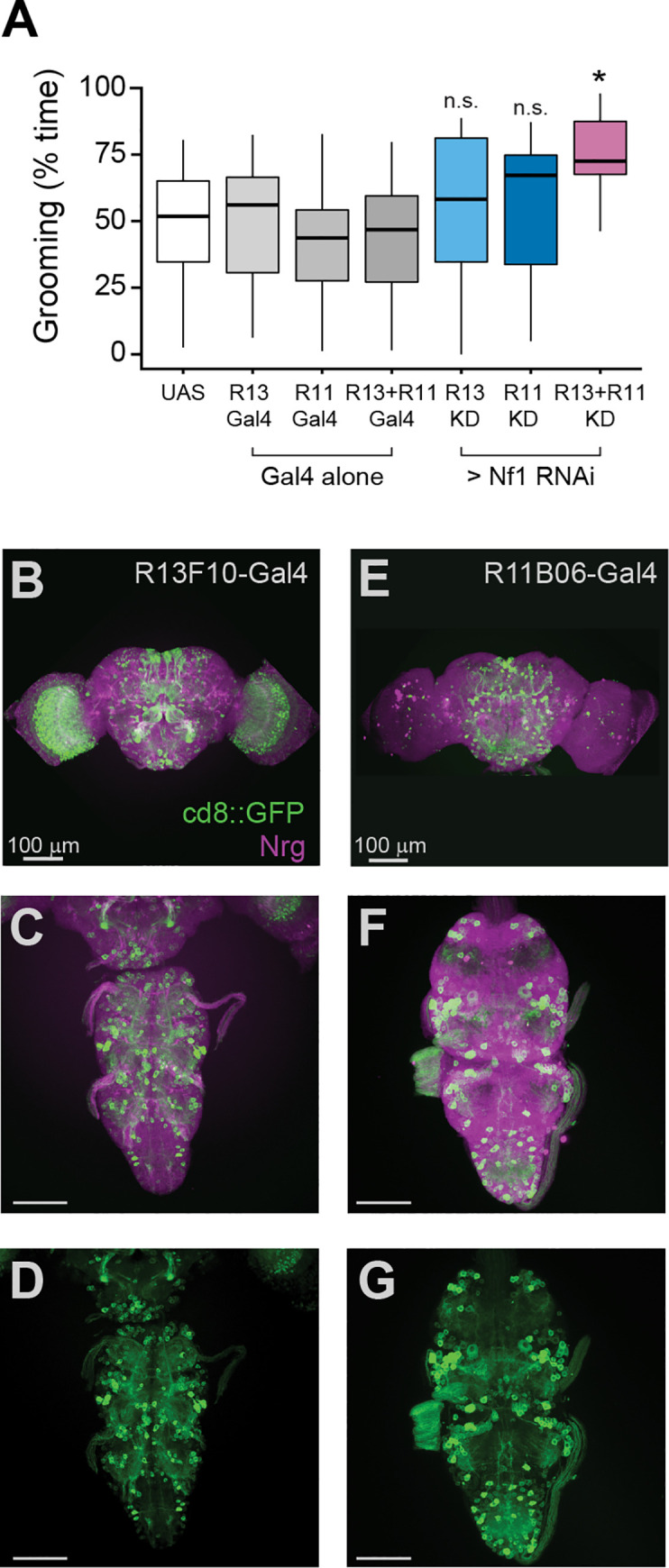
Loss of Nf1 across combinations of distributed circuits drives elevated grooming. (A) Grooming duration in flies with Nf1 knockdown in one or both sets of neurons labeled by the R13F10 (R13) and R11B06 (R11) Gal4 drivers. n = 20; Kruskal-Wallis; *p < 0.05 compared to UAS and Gal4 controls, n.s. not significant compared to UAS or Gal4 controls (Dunn/Sidak). (B) Maximum-intensity projection showing the expression of R13F10-Gal4 (green) and anti-neuroglian immunostaining (magenta) in a 48-hr pupal brain. (C) 48-hr pupal VNS, as in panel B. (D) GFP channel of VNS shown in panel C. (E) Maximum-intensity projection showing the expression of R11B06-Gal4 (green) and anti-neuroglian immunostaining (magenta) in a 48-hr pupal brain. (F) 48-hr pupal VNS, as in panel B. (G) GFP channel of the VNS shown in panel F.

To test whether subsets of neurons produced additive effects, we combined pairs of Gal4 drivers. Four pairs were selected (S2 Fig) based on their relatively high levels of grooming (albeit not significant relative to controls) and nonoverlapping expression patterns [52]. Among these, two pairs of Gal4 drivers, R13F10+R11B06 and R13F10+R42A08, significantly elevated grooming when combined to knockdown Nf1 (Fig 6A and S2 Fig). Midway through the pupal stage, R13F10 and R11B06 each label a sparse set of neurons that innervate the brain and VNS neuropil in a restricted pattern (Fig 6B–6D). Doubling the dosage of a single Gal4 driver (R13F10) did not increase grooming, suggesting that the addition of the new elements from the R11B06-Gal4 driver, rather than an increase in the Gal4 dosage within the same neurons (S2 Fig), produced the effect. Neuron-glia interactions are a possible cross-cell-type interaction that could produce effects on behavior. We tested this by combining knockdown the pan-glial driver repo-Gal4 with R13F10-Gal4, and this combination failed to produce significant grooming (S2 Fig). Overall, these data suggest that the requirement for Nf1 is distributed relatively broadly across neuronal circuits, with smaller subsets producing sub-threshold, additive effects.

## Discussion

The present results suggest that loss of Nf1 elevates grooming via effects during a developmental critical window, through actions that require the GAP-related domain, and via distributed cholinergic/oct-tyrR+ neurons. The requirement for Nf1 occurred during the pupal stage, suggesting that a distributed set of neurons may depend on Nf1 function during the formation and refinement of the adult nervous system. Oct-tyrR+ neurons are abundant during this developmental stage, though we did not explicitly test the developmental contributions of this neuronal subset in isolation. Several lines of evidence suggest that the requirement for Nf1 in order to maintain normal grooming levels is biased toward VNS neurons, including decapitation experiments, as well as tsh-Gal4/Gal80. One caveat is that the tsh-Gal4 driver exhibits expression in some neurons in the central brain (as well as the VNS). The decapitation experiments mitigate this caveat. While decapitated flies groom less than intact flies overall, decapitated *Nf1* mutants groom more than decapitated controls.

Development of the nervous system involves many important steps that are subject to plasticity, particularly during the pupal stage in *Drosophila*. During metamorphosis, neurons in the CNS exhibit dramatic changes, including programmed cell death, neurogenesis and proliferation, and neurite remodeling [54–56]. For example, connectivity between mushroom body Kenyon cells (KC) and presynaptic GABAergic neurons is dependent on pruning initiation from the post-synaptic γ KCs [57]. Losing Nf1 function could influence similar developmental processes in other parts of the nervous system and influence the development of grooming behaviors. Given the temporal resolution of the rescue experiments, our data do not rule out a dynamic role for Nf1 signaling in adult grooming, but strongly implicate developmental processes. Future studies into the developmental roles of Nf1 will be necessary to determine precisely how it influences the formation and function of neuronal circuits in the CNS, including those mediating grooming behaviors.

The requirement for intact GAP-related domain functionality suggests that Nf1-mediated Ras signaling is important for the development of normal grooming. As explored above, several critical neural developmental events occur during metamorphosis [54,55]. Ras signaling may affect these processes by acting downstream of receptor tyrosine kinases, some of which play key roles in development [58]. For instance, the receptor tyrosine kinase Alk and its ligand Jeb modulate multiple developmental processes, including patterning visceral muscle founder cells [59], mediating retinal axon targeting during metamorphosis [60], and shaping synaptic morphology and function at the neuromuscular junction [61,62]. Alk and Jeb are key genetic modifiers of the Nf1 body size/growth phenotype [63], and Nf1 mutants exhibit overgrown neuromuscular junction synapses [46]. Developmental effects of Ras have been particularly well-studied in the eye, where it modulates cell (photoreceptor) specification during metamorphosis [58,64]. Overall, this suggests that Nf1/Ras-related signaling is positioned to influence a range of developmental processes, particularly during metamorphosis.

We found that Nf1-sensitive neuronal elements modulating grooming were distributed and additive. Excessive grooming in Nf1 loss of function emanated from cholinergic (excitatory), oct-tyrR+ circuits, with VNS circuits playing a key role. The phenotype required knocking down Nf1 from across broad circuits, or combinations of smaller subsets. This contrasts with neuronal activation studies, in which some individual grooming behaviors have been mapped to small subcircuits, each driving grooming of a specific body part [31,53]. For instance, in the antennal grooming circuit, mechanosensory neurons innervate a command circuit consisting of layered excitatory and inhibitory neurons with varying reciprocal or feed-forward connections. Optogenetic stimulation of the excitatory neurons in this command circuit drives antennal grooming [31,35]. Other body part-specific command circuits likely exist at varying locations in the nervous system, particularly in the VNS, where the majority of peripheral somatosensory neurons provide input from the peripheral nervous system. We suggest that loss of Nf1 produces sub-threshold dysfunction across multiple grooming-involved neurons and that when enough of these neurons have lost Nf1, this additive influence results in behavioral deficits. We tested 212 Gal4 lines that label restricted sets of neurons for possible Nf1-dependent effects on grooming. None of these lines elevated grooming when used alone. Interestingly, we identified two pairs of drivers that elevated grooming when combined. Future studies will evaluate whether interactions between the identified pairs of circuits or the mere addition of Nf1 loss across a critical number of neurons are factors in the development of grooming hyperactivity.

The present data demonstrate a developmental, disease-relevant behavioral phenotype in a highly tractable genetic model organism. Neurodevelopmental disorders such as NF1 often involve complex alterations in cellular signaling and complex pathophysiology. These disorders emerge early in development and may involve both dysregulations of developmental processes and alterations in acute signaling/neuronal function in the developed nervous system. Nf1 has been observed to have important functions in adult mice and flies [14,19,27,28], and some phenotypes can be rescued by acute pharmacological treatment in adult animals [65]. Nonetheless, our data suggest that Nf1 loss disrupts a distributed set of microcircuits during a critical developmental window. Thus, Nf1 acts via pleiotropic mechanisms in the cellular signaling context (influencing broad signaling cascades including Ras and cAMP/PKA) to affect distributed neuronal circuits during development and adulthood. The *Drosophila* grooming model will enable future studies to pinpoint how the loss of Nf1 dysregulates distributed neuronal networks to produce behavioral deficits in adulthood.

## Materials and methods

### Fly strains

Flies were raised on cornmeal/agar food medium according to standard protocols. They were housed in incubators maintained at 25°C, 60% relative humidity, and kept on a 12:12 light:dark cycle. Male flies were used for all experiments. The *Nf1*^P1^ mutation was backcrossed for 6 generations into the *wCS10* genetic background. The *Nf1*^E1^ mutation was backcrossed for 6 generations into the *w;iso2;iso3* genetic background. In experiments with mixed genetic backgrounds (e.g., *Nf1*^P1^/*Nf1*^E1^), control groups were background matched. The R1320P mutation was created in a wild-type cDNA using the Q5 Site-Directed Mutagenesis Kit (New England Biolabs). Wild-type and R1320P mutant Nf1 were then subcloned into the pUAST-attB vector with an in-frame C342 terminal fusion with eGFP cDNA. Transgenic lines were produced by integrating the constructs at the attp40 site (Rainbow Transgenic Flies Inc.). The Nf1 RNAi line was obtained from the Vienna Drosophila RNAi Center (VDRC #109637) [36] and UAS-dicer2 was used in all crosses to enhance the RNAi effect. The empty attP control line (VDRC #60100) was used in Gal4/+ control crosses to ensure a matched genetic background across all groups. The following Gal4 lines were used in this study: nSyb-Gal4, elav-Gal4, and tub-Gal80^t.s.^ (gifts from Ronald L. Davis), tsh-Gal80 (gift from Julie Simpson), the following lines from the Bloomington *Drosophila* Stock Center (BDSC): TH-Gal4 (BDSC# 8848), 69B-Gal4 (1774), ChAT-T2A-Gal4 (60317), Gad1-Gal4 (51630), VGluT-T2A-Gal4 (60312), tsh-Gal4 (3040), R57C10-Gal4 (nSyb; 39171), R13F10 (48578), R11B06-Gal4 (48287), R42A08-Gal4 (50144), and other Gal4 lines from the FlyLight collection were used in the VNS screen (S1 Table). Immunostaining experiments were performed using flies expressing UAS-mCD8::GFP (BDSC #32195).

### Behavioral experiments

Grooming behavior experiments were performed as previously described [30]. The grooming chamber was an open field, 2.85 mm in height and 25.4 mm in diameter, consisting of an opaque (white) acrylic lateral boundary covered on the top and bottom with two clear polycarbonate sheets. The apparatus was illuminated from below with white LEDs that were filtered through a sheet of white acrylic; light intensity was measured at 720 lux in the location of the fly. Monochrome firefly MV 1394a or color firefly MV USB cameras fitted with Fujinon YV2.8×2.8SA-2 lenses were mounted above the arena. A single male fly was placed in each arena with an aspirator and recorded at 7.5 frames per second, 640 x 480 with Motion JPEG 2000 compression. Two or three videos were recorded for each subject, one immediately after loading into the chamber (0–5 min), a second at 5–10 minutes (in some experiments), and one at 15–20 min. Manual scoring of videos was carried out by an observer blind to the genotype. Start and stop frames were noted for each grooming event, which was further categorized according to which body part the fly was grooming: front legs, head/eye, abdomen, wings, or hind legs. The percentage of time spent grooming was calculated for either all events summed or individual body parts.

### Immunostaining

Nervous systems of pupae 0, 48, or 96 hours after pupal formation or adult flies 5–7 days old were dissected in 1% paraformaldehyde in S2 medium, and processed according to a published protocol [52]. Brains and ventral nervous systems were incubated with the primary antibodies for 3 hours at room temperature and at 4°C overnight, and with the secondary antibodies for 3 hours at room temperature and 4 days at 4°C. Incubations were performed in blocking serum (3% normal goat serum). Labeled brains were mounted in vectashield media. Antibodies used were rabbit anti-GFP (1:1000, Invitrogen), mouse anti-brp (nc82) (1:50, DSHB), mouse anti-neuroglian (1:50,DSHB), goat anti-rabbit IgG and goat anti-mouse IgG (1:800, Alexa 488 or Alexa 633 respectively, Invitrogen). Images were obtained using Leica TCS SP8 confocal microscope.

### Statistics

Normality of data was assessed with the Shapiro-Wilk Normality Test. Box plots graph the median as a line, the interquartile range (IQR) as a box, and whiskers extend to the largest value no further than 1.5xIQR; data beyond the whiskers are plotted individually as outliers. Hypothesis testing was carried out using the Wilcoxon rank-sum test (non-parametric) or Kruskal-Wallis omnibus test followed by Dunn’s test with Sidak correction for multiple comparisons (non-parametric). Two-way comparisons were carried out with a two-way ANOVA, followed by Tukey’s multiple comparisons tests. Statistics and graphing were performed with R version 3.2.3 with the ggplot2 package and Graphpad Prism.

## Supporting information

S1 Fig (A) Box plots showing grooming duration at the four indicated time points for flies with Nf1 knockdown using nSyb-Gal4 > Nf1 RNAi (nSyb>KD) or genetic controls with only UAS-Nf1. RNAi (UAS). n = 10; *, p < 0.05, ***, p < 0.001, Wilcoxon rank-sum test. (B) Box plots showing grooming duration for flies with Nf1 knockdown, scored at the 5–10 min time interval, with a variety of GAL4 drivers. Lines are ranked in order of ascending median grooming duration. A cutoff threshold of 40% for follow up evaluation is indicated by a dashed red line. Gal4s are listed in S1 Table.
(TIF)

S2 Fig Follow-up behavioral experiments for putative screen hits. All panels show box plots of grooming duration for the knockdown (KD) group compared to UAS- and Gal4-only or additional controls. Grooming was scored 15–20 min after introduction to chamber. (A-I) Individual Gal4 KD. n = 20; n.s., not significant, *p < 0.05, **p<0.01 (Dunn/Sidak). (J) An additive combination of R13F10-Gal4 and R11B06-Gal4 compared to UAS, R13F10-Gal4-only, heterozygotes of R13F10-Gal4 used for KD, homozygotes of R13F10-Gal4 used for KD, and a combination of R13F10-Gal4 with the pan-glial driver repo-Gal4. n = 20; in comparison to UAS and Gal4-only controls: n.s., not significant, ***p<0.001 (Dunn/Sidak). (K-L) Knockdown of Nf1 using an additive combination of R13F10-Gal4 with one other Gal4, compared to UAS, doubled Gal4s (2x Gal4), and single Gal4 knockdown controls. n = 20; in comparison to UAS and 2x Gal4-only controls: n.s., not significant, ***p<0.001 (Dunn/Sidak).
(TIF)

S1 Table Median and first and third quartiles for each Gal4 line used in the screen shown in S1 Fig. (XLSX)
