# Supplementary figures and images for: Developmental loss of neurofibromin across distributed neuronal circuits drives excessive grooming in Drosophila
Source: PLoS Genet. 2020 Jul 22;16(7):e1008920. doi: 10.1371/journal.pgen.1008920 (PMC7398555; doi:10.1371/journal.pgen.1008920)

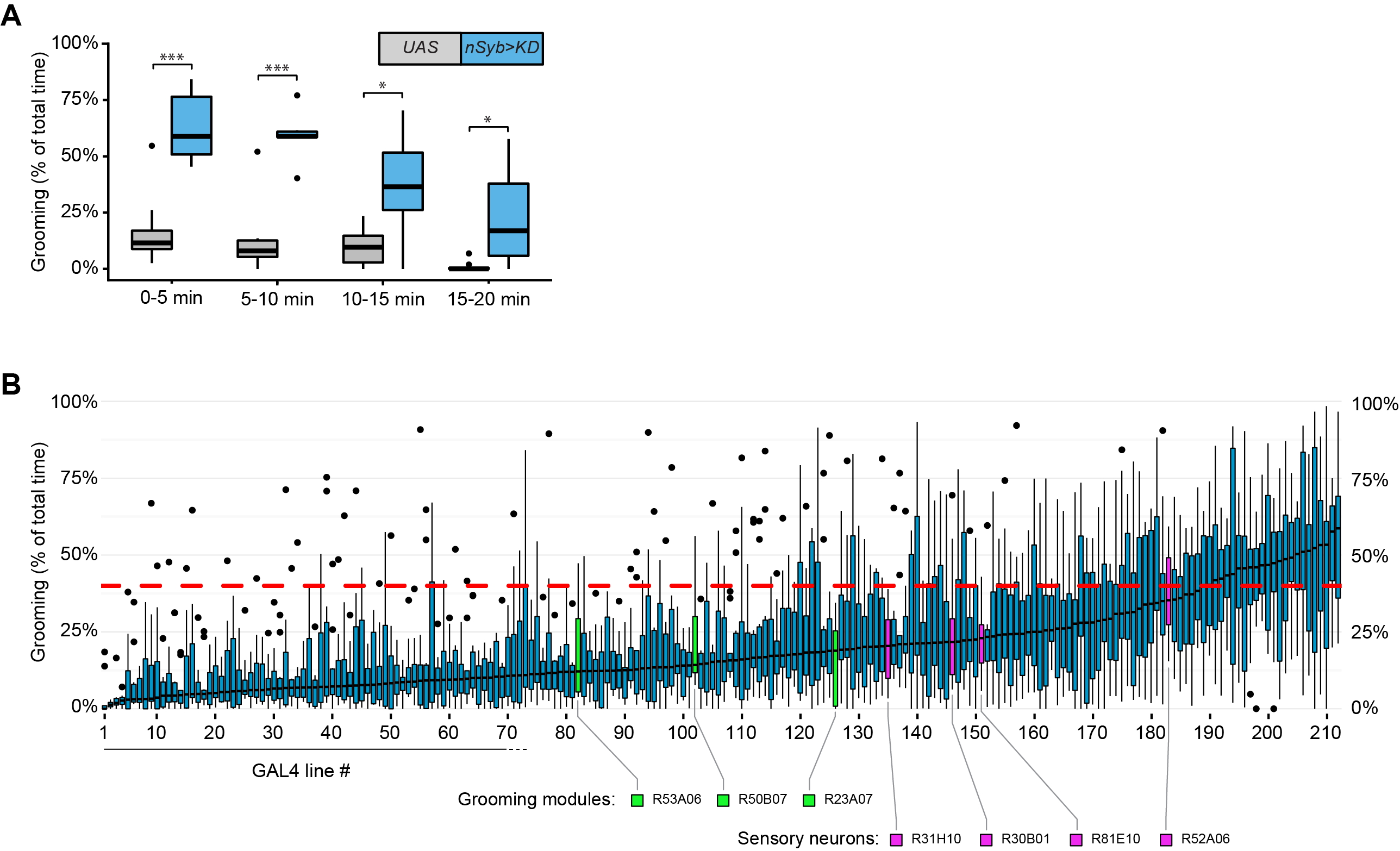

Supplement: S1 Fig — (A) Box plots showing grooming duration at the four indicated time points for flies with Nf1 knockdown using nSyb-Gal4 > Nf1 RNAi (nSyb>KD) or genetic controls with only UAS-Nf1. RNAi (UAS). n = 10; *, p < 0.05, ***, p < 0.001, Wilcoxon rank-sum test. (B) Box plots showing grooming duration for flies with Nf1 knockdown, scored at the 5–10 min time interval, with a variety of GAL4 drivers. Lines are ranked in order of ascending median grooming duration. A cutoff threshold of 40% for follow up evaluation is indicated by a dashed red line. Gal4s are listed in S1 Table. (TIF) [file pgen.1008920.s001.tif]

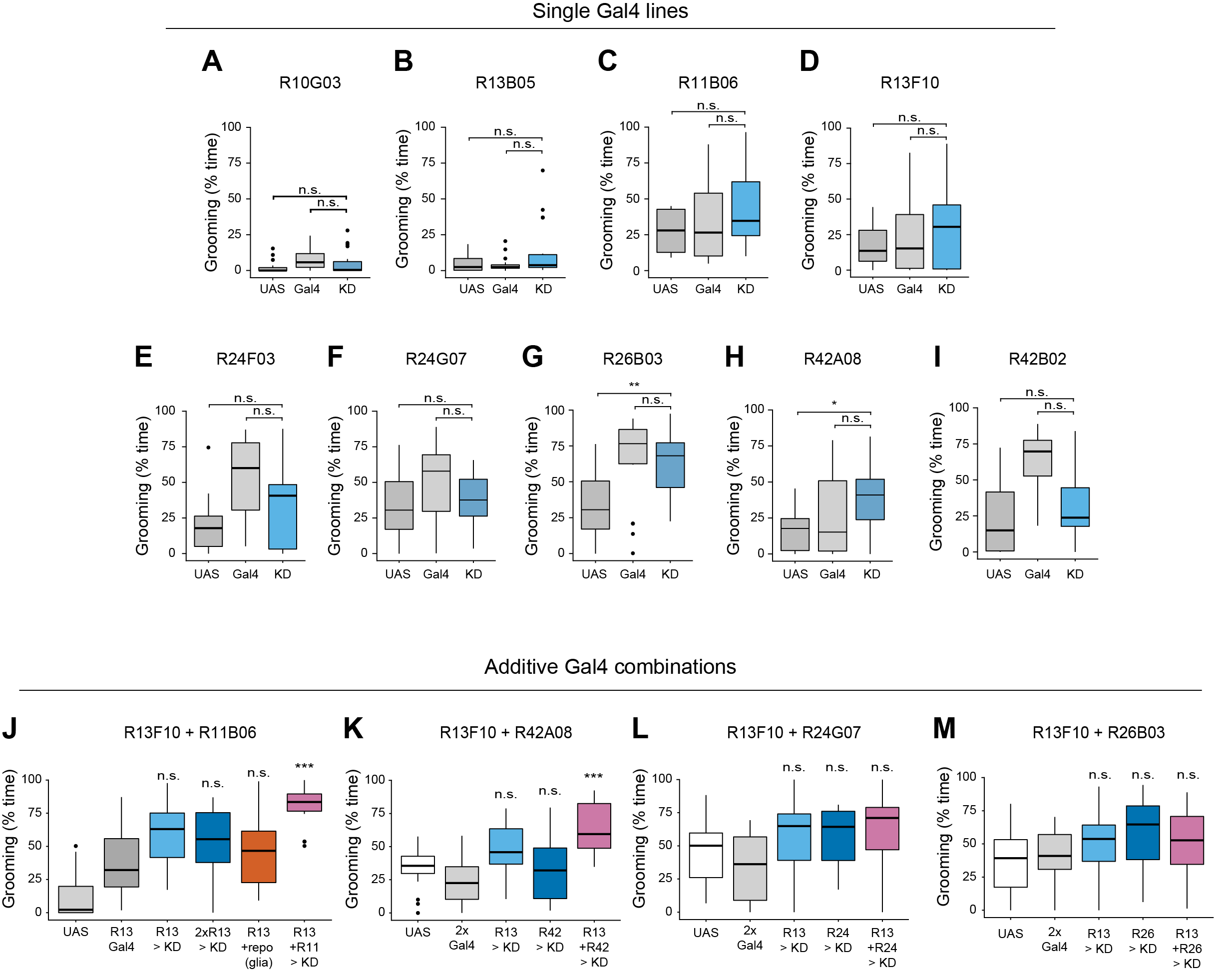

Supplement: S2 Fig — All panels show box plots of grooming duration for the knockdown (KD) group compared to UAS- and Gal4-only or additional controls. Grooming was scored 15–20 min after introduction to chamber. (A-I) Individual Gal4 KD. n = 20; n.s., not significant, *p < 0.05, **p<0.01 (Dunn/Sidak). (J) An additive combination of R13F10-Gal4 and R11B06-Gal4 compared to UAS, R13F10-Gal4-only, heterozygotes of R13F10-Gal4 used for KD, homozygotes of R13F10-Gal4 used for KD, and a combination of R13F10-Gal4 with the pan-glial driver repo-Gal4. n = 20; in comparison to UAS and Gal4-only controls: n.s., not significant, ***p<0.001 (Dunn/Sidak). (K-L) Knockdown of Nf1 using an additive combination of R13F10-Gal4 with one other Gal4, compared to UAS, doubled Gal4s (2x Gal4), and single Gal4 knockdown controls. n = 20; in comparison to UAS and 2x Gal4-only controls: n.s., not significant, ***p<0.001 (Dunn/Sidak). (TIF) [file pgen.1008920.s002.tif]
